# The Antimicrobial Peptide Esc(1-21) Synergizes with Colistin in Inhibiting the Growth and in Killing Multidrug Resistant *Acinetobacter baumannii* Strains

**DOI:** 10.3390/antibiotics11020234

**Published:** 2022-02-11

**Authors:** Federica Sacco, Camilla Bitossi, Bruno Casciaro, Maria Rosa Loffredo, Guendalina Fabiano, Luisa Torrini, Flavia Raponi, Giammarco Raponi, Maria Luisa Mangoni

**Affiliations:** 1 Department of Molecular Medicine, University of Rome “La Sapienza”, 00161 Rome, Italy; federica.sacco@uniroma1.it (F.S.); camilla.bitossi@uniroma1.it (C.B.); 2 Clinical Microbiology Laboratory, Sapienza University Hospital Policlinico Umberto I of Rome, 00161 Rome, Italy; flavia.raponi@yahoo.it; 3 Department of Biochemical Sciences, Laboratory Affiliated to Istituto Pasteur Italia-Fondazione Cenci Bolognetti, Sapienza University of Rome, 00185 Rome, Italy; bruno.casciaro@uniroma1.it (B.C.); mariarosa.loffredo@uniroma1.it (M.R.L.); fabiano.1524958@studenti.uniroma1.it (G.F.); torrini.1737960@studenti.uniroma1.it (L.T.); 4 Department of Public Health and Infectious Diseases, Sapienza University of Rome, 00185 Rome, Italy

**Keywords:** antibiotic resistance, *Acinetobacter baumannii*, antimicrobial peptides, colistin, synergy, membrane perturbation

## Abstract

Multidrug-resistant microbial infections and the scarce availability of new antibiotics capable of eradicating them are posing a serious problem to global health security. Among the microorganisms that easily acquire resistance to antibiotics and that are the etiological cause of severe infections, there is *Acinetobacter baumannii*. Carbapenems are the principal agents used to treat *A. baumannii* infections. However, when strains develop resistance to this class of antibiotics, colistin is considered one of the last-resort drugs. However, the appearance of resistance to colistin also makes treatment of the Acinetobacter infections very difficult. Antimicrobial peptides (AMP) from the innate immunity hold promise as new alternative antibiotics due to their multiple biological properties. In this study, we characterized the activity and the membrane-perturbing mechanism of bactericidal action of a derivative of a frog-skin AMP, namely Esc(1-21), when used alone or in combination with colistin against multidrug-resistant *A. baumannii* clinical isolates. We found that the mixture of the two compounds had a synergistic effect in inhibiting the growth and killing of all of the tested strains. When combined at dosages below the minimal inhibitory concentration, the two drugs were also able to slow down the microbial growth and to potentiate the membrane-perturbing effect. To the best of our knowledge, this is the first report showing a synergistic effect between AMPs, i.e., Esc(1-21), and colistin against colistin-resistant *A. baumannii* clinical isolates, highlighting the potential clinical application of such combinational therapy.

## 1. Introduction

Infections sustained by multi-drug-resistant bacteria (MDR), i.e., bacteria resistant to at least three different classes of antimicrobial agents, represent a serious threat for human health. *Acinetobacter baumannii* is a Gram-negative bacterium involved in the etiology of a wide range of clinical manifestations, including hospital-acquired pneumonia, urinary tract infections, and bloodstream and surgical site infections, which occur preferentially in patients admitted to intensive care units (where they have five to ten-fold higher risk of acquiring nosocomial infections) [1]. The colonization or infection sustained by *A. baumannii* has been invoked as an independent risk factor of mortality [2], though the debate on the direct attribution of mortality due to infections from *A. baumannii* is still open. Of paramount importance in this debate is the observation that a huge number of clinically isolated strains of *A. baumannii* are MDR [3]; carbapenems have been considered as appropriate agents for treatment of infections associated to these strains [4]. However, carbapenem resistance has been observed worldwide mostly due to the diffusion of several international bacterial clones [5]. Class D (OXA enzymes) carbapenemases have been identified as the main mechanism responsible for this resistance, although metalloenzymes are locally prevalent, especially in East Asia. The OXA carbapenemases of *Acinetobacter* spp. belong to four phylogenetic subgroups: OXA-23-like; OXA-24-like; OXA-51-like and OXA-58. OXA-51-like subgroups are intrinsic to *A. baumannii*, occurring in most or all of the strains [6]. The pan-European clones (lineages) I, II, or III, as defined by amplified fragment length polymorphism analysis [7] include many of these genotypes. The therapy of carbapenem-resistant *A. baumannii* infection often requires the use of colistin, available commercially as colistin sulfate for oral and topical use, and as colistimethate sodium for parenteral and aerosol therapy [8]. Despite the recent introduction of a new siderophore cephalosporin [9], colistin still represents one of the last therapeutic opportunities. Unfortunately, resistance to colistin has emerged worldwide [10], thus complicating the clinical management of *A. baumannii* infections. From this scenario, it is evident that the identification of new therapeutic strategies, as well as the elaboration of new classes of molecules able to counteract *A. baumannii* infections, is highly pressing. Given the lack of effective drugs treatment and the high cost of new antibiotic monotherapies, antibiotics combination provides a strong weapon to defeat antibiotic resistance [11,12,13,14,15]. A classic combinatorial drug therapy against Gram-negative bacteria includes β-lactams plus aminoglycosides. However, despite the promising results shown by this combination either from in vitro or from animal studies, clinical data have been contrasting [16,17,18,19]. Gene-encoded antimicrobial peptides (AMPs) of the innate immunity are attractive candidates for alternative anti-infective compounds. These are short-length and low molecular weight molecules with interesting properties such as (i) broad spectrum of antimicrobial activity; (ii) membrane-perturbing effect as a primary mechanism of antibacterial action that confers cell selectivity limiting the induction of resistance; (iii) capability to modulate the host immune response and to promote wound-healing; and (iv) ability to synergize with other AMPs or conventional antibiotics [20,21,22,23,24]. We have previously characterized a 21-residues long derivative of the frog-skin AMP esculentin-1a, namely Esc(1-21). It is active against Gram-positive and Gram-negative bacteria, and it is endowed with the capacity to kill the human pathogen *Pseudomonas aeruginosa* and to enhance the growth inhibition activity of aztreonam [22,25,26]. In this work, with the aim to re-evaluate the efficacy of colistin, we investigated whether the usage of this drug in combination with Esc(1-21) had a synergistic effect in inhibiting the growth and/or in killing colistin-resistant clinical *A. baumannii* strains along with the underlying molecular mechanism. 

## 2. Results

Four strains (#1, #2, #3, #4) of *A. baumannii* were isolated from different clinical samples. We initially evaluated their susceptibility to colistin and to Esc(1-21), by the broth microdilution assay to determine the minimal inhibitory concentration (MIC). As indicated in Table 1, the MIC of colistin was 8 mg/L for the #3 strain, 16 mg/L for the #1 and #4 strains, and 32 mg/L for the #2 strain. This latter also presented the growth of colonies heteroresistant to colistin, as verified by MIC test strip assay. The MIC of Esc(1-21) was 17.5 mg/L for the #1, #3, and #4 strains, while it was 35 mg/L for the #2 strain (Table 1).

All isolates showed the intrinsic *bla*_OXA-51_ gene, confirming the taxonomic assignment as *A. baumannii*, and *bla*_OXA-23_ gene. The three isolates #1, #3, and #4 were assigned to sequence group (SG) 1 and associated with international clones (ICL) II, as they amplified genes of length equal to 580 bp (*csuE*), 162 bp (*bla*_OXA-51_), and 343 bp (*ompA*); only the #2 strain belonged to the SG4, which has not yet been related to any clonal lineage. 

Subsequently, the checkerboard assay was used to identify the possible existence of a synergistic antimicrobial activity by the combination of the two antimicrobials in comparison to the individual activities. As reported in Table 1, the MIC of both compounds when used in combination (MIC_FIC_) were lower than the corresponding MICs when tested alone, giving a FIC value ranging from 0.25 to 0.37 for all of the selected strains. This proves the occurrence of a strong synergism between the two compounds in inhibiting the growth of all *A. baumannii* strains.

**Figure 1:**
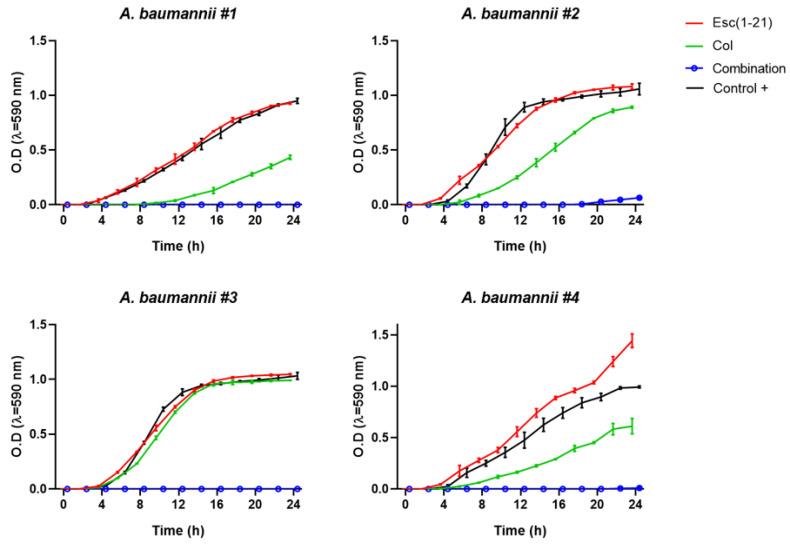
Growth profiles for the #1, #2, #3, and #4 *A. baumannii* strains treated with colistin (Col), Esc(1-21), or their combination at the MIC_FIC._ Untreated control samples were included for comparison. Results are of the mean ± standard deviation (SD) of three independent experiments.

The bacterial growth kinetics of the four strains are shown in Figure 1. When the two compounds were mixed at the MIC_FIC_, there was no increase in optical density for strains #1, #3, and #4 within 24 h indicating a complete inhibition of bacterial growth, while for strain #2 a slight increase of optical density was recorded above 18 h. 

**Figure 2:**
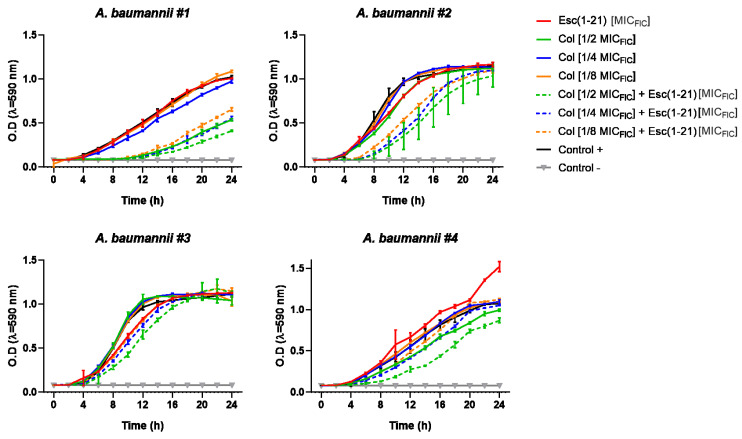
Growth kinetics for the different *A. baumannii* strains in the presence of colistin (Col, at its 1/2, 1/4 and 1/8 MIC_FIC_), Esc(1-21) (at its MIC_FIC_) and their combinations. Results are of the mean ± SD of three independent experiments.

The antimicrobial efficacy of colistin-Esc(1-21) mixture was also observed when Esc(1-21) was used at its MIC_FIC_ in combination with 1/2, 1/4 and 1/8 MIC_FIC_ of the antibiotic. Indeed, as reported in Figure 2, absorbance of samples along the time clearly pointed out that the addition of the peptide to different concentrations of colistin slowed down the microbial growth rate of all strains (dotted line) compared to what was found for the two drugs when used separately (full line).

To further investigate whether the antimicrobial activity of the drugs combination was accompanied by a bactericidal effect, aliquots from samples containing the two drugs at their MIC_FIC_ were withdrawn, 24 h after incubation at 37 °C, and seeded on agar plates for colonies counting. Interestingly, no viable bacterial cells were obtained for #1, #3, and #4 strains indicating that combination of the two compounds at their MIC_FIC_ had a complete bactericidal activity. In comparison, this effect was less pronounced for strain #2, with a killing activity of ~99%.

**Figure 3:**
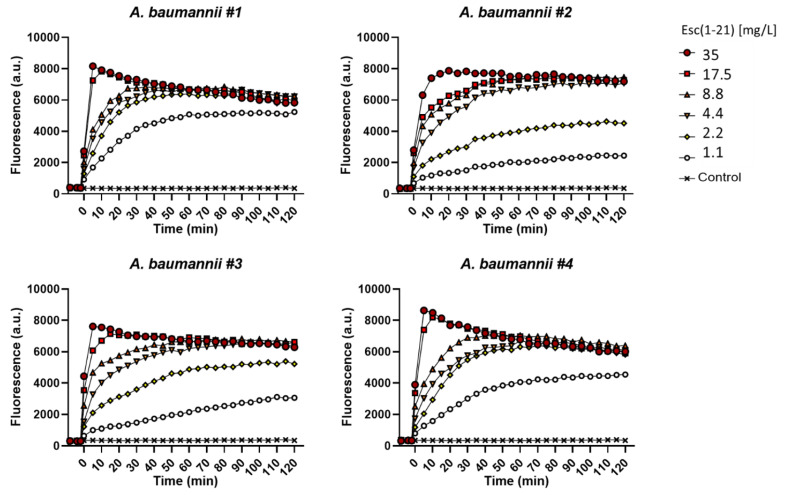
Kinetics of membrane permeabilization of all *A. baumannii* strains induced by the addition of Esc(1-21) (t = 0) at different concentrations. Samples were incubated with 1 μM Sytox Green (SG) in phosphate buffer saline and changes in fluorescence were monitored. Controls were microbial cells with vehicle. Values correspond to one representative experiment of three.

As already demonstrated for other bacterial strains, one of the primary antimicrobial mechanisms of Esc(1-21) is the perturbation of the cytoplasmic membrane. To verify whether this mechanism was preserved against *A. baumannii* species and to characterize the kinetics of membrane perturbation, the membrane-impermeable fluorescent probe Sytox Green was employed. Its fluorescence intensity significantly increases upon binding to nucleic acids once it has entered cells with a damaged membrane. As shown in Figure 3, for all *A. baumannii* strains, a fast and dose-dependent membrane perturbation process was induced by Esc(1-21). This is manifested by the rapid increase of fluorescence intensity within the first minutes of peptide addition (time = 0, Figure 3).

**Figure 4:**
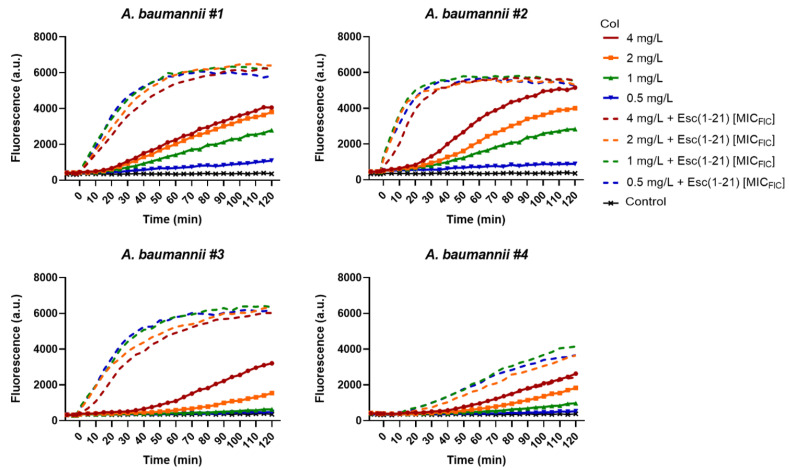
Kinetics of membrane permeabilization of all *A. baumannii* strains induced by the addition of different concentrations of colistin (Col), either when used alone or in combination with Esc(1-21) at its MIC_FIC_. Samples were incubated with 1 μM Sytox Green in phosphate buffer saline as described in the Materials and Methods section, and changes in fluorescence were monitored. Controls were microbial cells with vehicle. Values correspond to one representative experiment of three.

The exact mechanism of action of the cationic colistin is still unclear, but the primary microbicidal effect is believed to be triggered by its electrostatic interaction with the negatively-charged lipid A of lipopolysaccharides (LPS), the major components of the outer membrane in Gram-negative bacteria. When the permeabilization of the outer membrane occurs, colistin translocates through it in a self-promoted manner, and subsequently provokes the destabilization of the inner membrane with consequent cell death. As reported in Figure 4, the membrane-perturbing activity of colistin (full lines) was slower than that of Esc(1-21) which is shown in Figure 3. Interestingly, when Esc(1-21), at its MIC_FIC_, was added to concentrations of colistin ≤MIC i.e., 4, 2, 1 and 0.5 mg/L (dotted lines), the kinetics of membrane perturbation became faster with a similar profile to that of the frog-skin AMP at the corresponding concentration (i.e., 2.2 mg/L against strain #1, 4.4 mg/L against strains #2 and #3, and 1.1 mg/L against strain #4).

## 3. Discussion

As a polymyxin antibiotic, colistin is a cationic polypeptide that exerts its antibacterial effect against a wide range of Gram-negative bacteria. Although the exact mechanism of bacterial killing of polymyxins is not clearly defined, colistin affects the permeability of the LPS-outer membrane through the electrostatic interaction with the negatively-charged lipid A [27]. Colistin represents one of the main therapeutic options in treating infections sustained by carbapenem-resistant-MDR *A. baumannii* strains, but unfortunately resistance to it has frequently been described [5]. Colistin resistance generally implies a lower binding affinity to LPS due to a reduced anionic character of LPS. This can be the consequence of different mechanisms: (i) mutations in the *pmrCAB* locus which regulates the phosphoethanolamine (pEtN) transferase with replacement of phosphate groups of lipid A by phosphoethanolamine; (ii) impairment of LPS synthesis by *ramA* locus mutations; (iii) activation of efflux pumps such as KpnEF, AcrAB, and Sap proteins systems [28,29]. *A. baumannii* is nowadays considered one of the most relevant clinical microorganisms, being the etiological agent of a large variety of nosocomial infections, encompassing bacteremia, meningitis, pneumonia, and urinary tract infections [28,30,31]. The spread of genes encoding for oxacillinases (*bla*_OXA-like_) promoted carbapenem resistance in *A. baumannii* isolates. Currently, OXA-58 oxacillinases have drastically decreased in Italy, being replaced by OXA-23 [31]. The *A. baumannii* strains analyzed in our work, all phenotypically resistant to carbapenem as well as to colistin (MIC > 4 mg/L), were consistent with the MDR *A. baumannii* strains circulating in Italy in the last decade, carrying the *blaOXA-23* carbapenemase gene: three isolates (i.e., #1, #3, and #4) were related to ICL II, while #2 strain belonged to SG4, which is not related to any ICL. Due to multiple resistance mechanisms, involving β-lactamases production, enhanced expression of efflux pumps, alteration of target sites of conventional antibiotics, and decreased membrane permeability, *A. baumannii* causes infections that make even last-line antibiotics or antibiotic-combination therapies almost ineffective.

AMPs constitute a valid alternative against multidrug-resistant bacteria either if used alone or in combination with traditional antibiotics [32]. In the light of AMPs’ properties, several studies have already proposed a beneficial effect from the combination of AMPs and antibiotics, especially in the case of MDR and biofilm-forming organisms [33,34]. More precisely, in recent years, several groups have investigated the combinatory effect of AMPs and antibiotics against MDR *A. baumannii*. As an example, Jangra and coworkers [35] tested the AMP Tridecaptin M together with rifampicin, vancomycin, clarithromycin, imipenem, and ceftazidime against several MDR *A. baumannii* strains and obtained FIC values ranging from 0.25 to 0.5 [35]. Jahangiri et al. [36], proved that combination of the polycyclic peptide nisin with colistin gives rise to an additive effect against the resistant strains of *A. baumannii* used in their study, except for one strain against which a synergistic effect was observed (FIC = 0.5) [36]. Lately, Witherell and coworkers [37] showed a synergistic activity between the AMP CDP-B11 and colistin against several MDR Gram-negative bacteria, including *A. baumannii*. However, this strain had only an intermediate resistance to colistin (MIC = 1 mg/L) [37]. To the best of our knowledge, our work is the first demonstration of the synergistic effect between the short linear cationic AMP (i.e., Esc(1-21) and colistin), against colistin-resistant *A. baumannii* (MIC of colistin > 4 mg/L). We previously discovered an additive effect of Esc(1-21) in inhibiting the growth of the Gram-negative bacterium *Pseudomonas aeruginosa* when combined with aztreonam and hypothesized that Esc(1-21) served as helper agent to facilitate the intracellular influx of the antibiotic, allowing it to exert the toxic effect on bacteria, by blocking peptidoglycan crosslinking [38]. In the case of colistin, which mainly acts on the bacterial outer membrane, the addition of Esc(1-21) would contribute to potentiate the membrane perturbing activity of the peptides, thus leading to microbial death at concentrations lower than those needed by the single compounds. Overall, our experiments have clearly highlighted that Esc(1-21) is able to synergize with colistin in killing four strains of MDR *A. baumannii*, despite the fact that this effect was more limited versus the SG4 strain. 

Although AMPs have been found to be active against colistin-resistant *A. baumannii* [39,40,41], studies on their efficacy in inhibiting and/or in killing these strains when combined with colistin are very limited. Remarkably, the combination of AMP plus colistin holds great promise; Otvos and colleagues demonstrated that the combination of the peptide A3-APO with colistin reduces the effective dose of this latter also in a bacteremia mouse model of *Klebsiella pneumoniae* [42]. Overall, beside showing the effectiveness of Esc(1-21) in potentiating the antimicrobial effect of colistin by likely accelerating the membrane perturbation of *A. baumannii* cells, our data have emphasized the possibility to re-evaluate the last resort antibiotic colistin, thus opening new avenues based on a combinatorial AMP/antibiotic-based therapy for treatment of MDR *A. baumannii* infections resistant to colistin.

## 4. Materials and Methods

### 4.1. Materials

The AMP Esc(1-21) was purchased from Biomatik (Wilmington, DE, USA; Kitchener, ON, Canada). It was assembled by stepwise solid-phase synthesis and purified via reverse-phase high-performance liquid chromatography to a purity of 98% using a gradient of acetonitrile in 0.1% aqueous trifluoroacetic acid (from 28% to 100% in 30 min) at a flow rate of 1.0 mL/min. The molecular mass was verified by electron spray ionization mass spectrometry. Colistin sulfate was purchased from Sigma-Aldrich (Milan, Italy).

### 4.2. Microbial Strains

The strains of *A. baumannii* (n = 4) used in the study were isolated from four patients admitted to the Intensive Care Unit of a 1300-bed tertiary care academic hospital (Policlinico “Umberto I”, Rome, Italy). The strains #1 and #4 were isolated from blood cultures, while the strains #2 and #3 were isolated from respiratory secretions. The samples were cultured on agar media (Columbia 5% blood Agar and MacConkey Agar, BioMérieux, Marcy l’Etoile, France) incubated for 24 h at 37 °C. The bacterial colonies were identified by MALDI-TOF mass spectrophotometry (Bruker Daltonics, Bremen, Germany), with a discriminatory score > 2300 [43].

Since carbapenem-resistant isolates are producing oxacillinases enzymes belonging to molecular class D (OXA enzymes), multiplex PCR was performed to identify resistance genes belonging to oxacillinases (*bla*_OXA-like_). The sequences of *bla*_OXA-like_ alleles encoding carbapenemases were aligned and group-specific regions were identified using Bioedit software [44]. Primer pair 5′-TAA TGC TTT GAT CGG CCT TG and 5′-TGG ATT GCA CTT CAT CTT GG was used to amplify a 353 bp fragment of genes encoding the intrinsic OXA-51-like enzymes of *A. baumannii*. These primers were combined with six new primers that were designed to amplify fragments of genes encoding acquired OXA-23-like (501 bp: 5′-GAT CGG ATT GGA GAA CCA GA and 5′-ATT TCT GAC CGC ATT TCC AT), OXA-24-like (246 bp: 5′-GGT TAG TTG GCC CCC TTA AA and 5′-AGT TGA GCG AAA AGG GGA TT) and OXA-58-like (599 bp: 5′-AAG TAT TGG GGC TTG TGC TG and 5′-CCC CTC TGC GCT CTA CAT AC), OXA-143-like (149 bp: 5′-TGG CAC TTT CAG CAG TTC CT and 5′-TAA TCT TGA GGG GGC CAA CC) carbapenemases (Table 2, Supplementary Figure S1). The amplification conditions were initial denaturation at 94 °C for 5 min, 30 cycles of 94 °C for 25 s, 52 °C for 40 s, and 72 °C for 50 s, and a final elongation at 72 °C for 6 min [45,46].

A typing scheme based on two multiplex PCRs targeting three genes under selective pressure (*ompA*, *csuE*, and *bla*_OXA-51-like_) was used for rapid assignment of *A. baumannii* isolates into three major PCR-based groups (Gs) corresponding to international clones I (G2), II (G1), and III (G3) [47]. Multiplex PCRs for identification of the *ompA*, *csuE*, and *bla*_OXA-51-like_ sequence groups defined as Group 1 and Group 2 were performed using the primers listed in Table 3 (Supplementary Figure S2).

Primers for *ompA* and *csuE* were designed from sequences available at GenBank (AY485227, DQ093960; AY241696) or from sequences determined during initial studies (DQ289014–DQ289019). These were aligned and consensus primers were designed from common sequence areas. The amplification conditions were: 94 °C for 3 min, followed by 30 cycles of 94 °C for 45 s, 57 °C for 45 s, and 72 °C for 1 min, followed by a final extension at 72 °C for 5 min [47,48]. Identification of a strain as a member of Group 1 or Group 2 required the amplification of all three fragments in the corresponding multiplex PCR, and an absence of any amplification by the other multiplex PCR. Group 3 isolates were defined by the amplification of only the *ompA* fragment in the Group 2 PCR, and the amplification of only the *csuE* and *bla*_OXA-51-like_ fragments in the Group 1 PCR.

### 4.3. Antimicrobial Assays

Antimicrobial susceptibility was performed by broth microdilution assay using Microscan Walkaway System (Beckman Coulter Inc, Brea, CA, USA) following the manufacturer’s instructions. The minimal inhibitory concentration (MIC) for colistin and Esc(1-21) was assessed by serial twofold dilutions in cation-adjusted Mueller-Hinton II broth (CAMHB) in a manual broth microdilution method, carried out by inoculating 100 µL of a bacterial suspension (5 × 10^5^ CFU/mL) in the wells of a sterile round bottom polystyrene microplate (Thermo Scientific, Denmark) and incubated at 37 °C for 24 h. The latest EUCAST interpretative criteria of the MICs for colistin were used, considering as resistant the strains with an MIC ≥ 4 mg/L [49]. The presence of colistin heteroresistance was checked by plating the bacterial suspension at 0.50 MF and 2 MF on Mueller–Hinton Agar II plates and tested with MIC Test Strips at 37 °C for 48 h.

The checkerboard titration method was used for the assessment of the synergistic activity of colistin in combination with Esc(1-21), following the CLSI guidelines [50,51]. Briefly, antimicrobial stock solutions were diluted in the microtiter plates to reach 8 × MIC of colistin and 4 × MIC Esc(1-21), and thereafter inoculated with each *A. baumannii* isolate at 0.50 MF turbidity in brain heart infusion broth (Microbiol, Cagliari, Italy). The interaction of colistin (A) with Esc(1-21) peptide (B) was evaluated by the fractional inhibitory concentration index (ΣFIC). This index was calculated according to the equation: ΣFIC = FIC_A_ + FIC_B_ = (MIC_A_ in combination/MIC_A_ alone) + (MIC_B_ in combination/MIC_B_ alone). According to the National Committee for Clinical Laboratory Standards (NCCLS) guidelines, the combination was considered synergistic when the ΣFIC is <0.5, indifferent when the ΣFIC is ≥0.5 to <2, and antagonistic when the ΣFIC is ≥2. 

The growth dynamics of the four *A. baumannii* strains in the presence of colistin and Esc(1-21) scalar concentrations was also tested kinetically in sterile microplates using a microtiter plate reader (BioTek Instruments, Inc., Winooski, VT, USA) as previously described for the checkerboard assay. The sample absorbance was automatically detected at OD_590_ at two-hour intervals for 24 h at 37 °C. The results were used to construct the bacterial growth curves, correlating to the absorbance in function of time. In addition, to evaluate a possible killing effect, aliquots of 30 µL, drawn from the FIC wells, were seeded on Mueller-Hinton II agar plates (BioMérieux, Marcy l’Etoile, France) and incubated at 37 °C for 24 h.

### 4.4. Membrane Permeabilization: Sytox Green Assay

To assess the ability of Esc(1-21) and colistin to perturb the cytoplasmic membrane permeability of *A. baumannii* when used alone or in combination, the Sytox Green assay was performed as previously reported [25]. Approximately 1 × 10^7^ CFU/mL were incubated with 1 μM Sytox Green in PBS for 5 min in the dark. After peptide addition, changes in fluorescence intensity (λexc = 485 nm, λems = 535 nm) caused by the binding of the dye to intracellular DNA were monitored for 120 min in a microplate reader (Infinite M200, Tecan, Salzburg, Austria) at 37 °C. Controls were cells not treated with the peptides.

## 5. Conclusions

Our data clearly showed that the combination of the AMP Esc(1-21) and colistin have a synergistic effect in inhibiting the growth and in killing colistin-resistant *A. baumannii* clinical isolates, probably through the potentiation of the membrane-perturbing activity of the compounds. The potential clinical application of such combination therapy should be further evaluated.

## Figures and Tables

**Table 1:** MICs and combinatory effect of colistin (Col) and Esc(1-21) against the four *A. baumannii* strains and relation to clonal lineage.

Isolate id.	ICL (SG)	Molecules	MICs Alone *	MICs in Combination (MIC_FIC_) *	ΣFIC
#1	II (SG1)	Col	16	4	0.37
		Esc(1-21)	17.5	2.2	
#2	na, (SG4) ^§^	Col	32	4	0.25
		Esc(1-21)	35	4.4	
#3	II (SG1)	Col	8	1	0.37
		Esc(1-21)	17.5	4.4	
#4	II (SG1)	Col	16	4	0.31
		Esc(1-21)	17.5	1.1	

na, international clones (ICL) not assigned; ^§^ Heteroresistant isolate; * [mg/L].

**Table 2:** Primers used for PCR amplification of oxacillinase resistance genes (*bla*_OXA-like_).

Primer	Sequence (5′-3′)	Target	Amplicon Size (bp)	References
*bla*_OXA-51_ FW	TAA TGC TTT GAT CGG CCT TG	*bla*_OXA-51-like_	353	[45,46]
*bla*_OXA-51_ RV	TGG ATT GCA CTT CAT CTT GG			
*bla*_OXA-23_ FW	GAT CGG ATT GGA GAA CCA GA	*bla*_OXA-23-like_	501	
*bla*_OXA-23_ RV	ATT TCT GAC CGC ATT TCC AT			
*bla*_OXA-24_ FW	GGT TAG TTG GCC CCC TTA AA	*bla*_OXA-24-like_	246	
*bla*_OXA-24_ RV	AGT TGA GCG AAA AGG GGA TT			
*bla*_OXA-58_ FW	AAG TAT TGG GGC TTG TGC TG	*bla*_OXA-58-like_	599	
*bla*_OXA-58_ RV	CCC CTC TGC GCT CTA CAT AC			
*bla*_OXA-143_ FW	TGG CAC TTT CAG CAG TTC CT	*bla*_OXA-143-like_	149	
*bla*_OXA-143_ RV	TAA TCT TGA GGG GGC CAA CC			

**Table 3:** Primers used in multiplex PCRs for identification of international clonal lineages.

Primer	Sequence (5′-3′)	Target	Amplicon Size (bp)	References
Group1ompAF306	GAT GGC GTA AAT CGT GGT A	*ompA*	355	[47,48]
Group1and2ompAR660	CAA CTT TAG CGA TTT CTG G			
Group1csuEF	CTT TAG CAA ACA TGA CCT ACC	*csuE*	702	
Group1csuER	TAC ACC CGG GTT AAT CGT			
Gp1OXA66F89	GCG CTT CAA AAT CTG ATG TA	*bla*_OXA-51-like_	559	
Gp1OXA66R647	GCG TAT ATT TTG TTT CCA TTC			
Group2ompAF378	GAC CTT TCT TAT CAC AAC GA	*ompA*	343	
Group1and2ompAR660	CAA CTT TAG CGA TTT CTG G			
Group2csuEF	GGC GAA CAT GAC CTA TTT	*csuE*	580	
Group2csuER	CTT CAT GGC TCG TTG GTT			
Gp2OXA69F169	CAT CAA GGT CAA ACT CAA	*bla*_OXA-51-like_	162	
Gp2OXA69R330	TAG CCT TTT TTC CCC ATC			

## Data Availability

Data is contained within the article or supplementary material.

## Supplementary Materials

The following supporting information can be downloaded at: https://www.mdpi.com/article/10.3390/antibiotics11020234/s1. Figure S1: Multiplex PCR performed on the 4 strains (#1–#4) of *A. baumannii* selected in this study for the assignment of the clonal lines to which they belong. The multiplex PCR was performed for the amplification of specific fragments within the *ompA*, *csuE*, *bla*OXA-51 genes, according to the primers combinations (SG1 and SG2) described in the materials and methods; Figure S2: Multiplex PCR performed on the 4 strains (#1–#4) of *A. baumannii* selected in this study for the identification of OXA genes. Multiplex PCR was performed for amplification of specific fragments within different variants of OXA genes. The band corresponding to the *bla*OXA-23 and *bla*OXA-51 genes is shown in the figure.
