# Supplementary material for: The Antimicrobial Peptide Esc(1-21) Synergizes with Colistin in Inhibiting the Growth and in Killing Multidrug Resistant Acinetobacter baumannii Strains
Source: Antibiotics (Basel). 2022 Feb 11;11(2):234. doi: 10.3390/antibiotics11020234 (PMC8868345; doi:10.3390/antibiotics11020234)
Supplement: Supplementary file 1 [file antibiotics-11-00234-s001.zip › antibiotics-1577068-supplementary.pdf]

Supplementary Materials

The Antimicrobial Peptide Esc(1-21) Synergizes with Colistin in Inhibiting the Growth and in Killing Multidrug Resistant *Acinetobacter baumannii* Strains

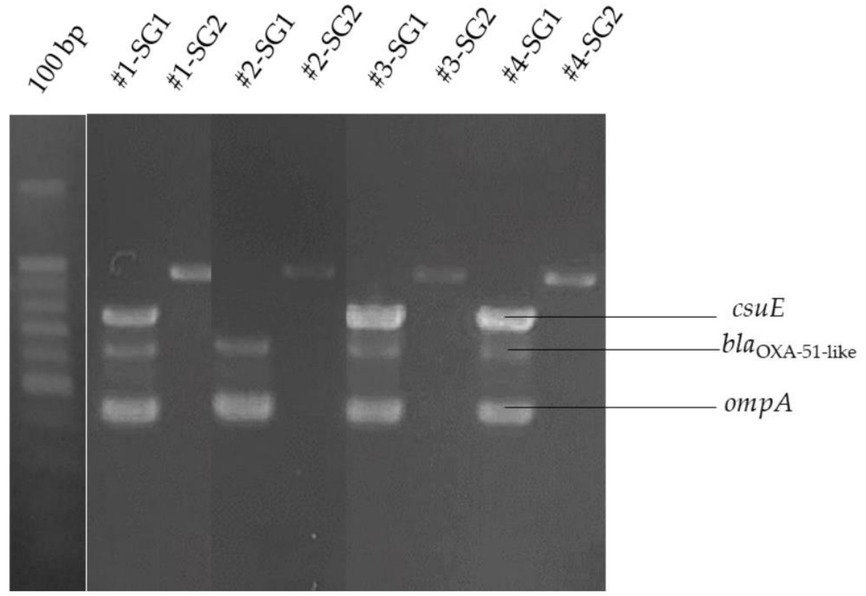

Figure S1. Multiplex PCR performed on the 4 strains (#1-#4) of *A. baumannii* selected in this study for the assignment of the clonal lines to which they belong. The multiplex PCR was performed for the amplification of specific fragments within the *ompA*, *csuE*, *bla*$_{OXA-51}$ genes, according to the primers combinations (SG1 and SG2) described in the materials and methods.

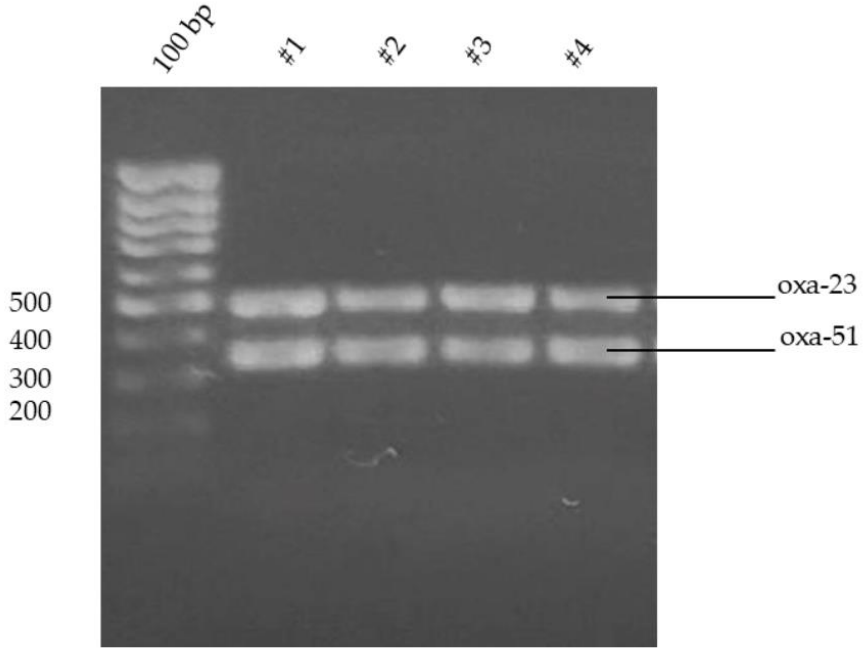

Figure S2. Multiplex PCR performed on the 4 strains (#1-#4) of *A. baumannii* selected in this study for the identification of OXA genes. Multiplex PCR was performed for amplification of specific fragments within different variants of OXA genes. The band corresponding to the *bla*$_{OXA-23}$ and *bla*$_{OXA-51}$ genes is shown in the figure.
